# miR-3064-5p and miR-4745-5p affect heparin sensitivity in patients undergoing cardiac surgery by regulating AT-III and factor X mRNA levels

**DOI:** 10.3389/fphys.2022.914333

**Published:** 2022-08-12

**Authors:** Hai-Ping Ma, Min Fu, Maisitanguli Masula, Chang-Shuang Xing, Qiang Zhou, Jing-Tong Tan, Jiang Wang

**Affiliations:** The First Affiliated Hospital of Xinjiang Medical University, Urumqi, China

**Keywords:** heparin, cardiopulmanory bypass, Antithrombin 3 (AT-III), Factor X (FX), miRNA, microRNA

## Abstract

**Subject:** Perioperative regulation of coagulation function through heparin in patients undergoing cardiac surgery with cardiopulmonary bypass is an important part of performing cardiac surgery, and postoperative bleeding due to abnormal coagulation function caused by differences in heparin sensitivity in different individuals is an independent risk factor for postoperative complications and death.

**Method:** Using an online database, 10 miRNAs interacting with AT-III and FX genes were predicted. Patients were divided into three groups according to the difference in activated clotting time (ACT) after the first dose of heparin (2.5 mg kg^−1^): group A: hyposensitive group (ACT < 480 s); group B: sensitive group (480 s ≤ ACT ≤ 760 s); and group C: hypersensitive group (ACT > 760 s). Perioperative and 24 h postoperative blood loss and other clinical data of patients in the three groups were recorded. Blood samples were collected before surgery, and RT-PCR was used to detect the levels of AT-III and FX gene mRNA and the levels of predicted 10 miRNAs.

**Result:** Heparin sensitivity was positively correlated with AT-III mRNA levels and negatively correlated with FX gene mRNA levels in the three groups, and the blood loss in group B was significantly lower than that in groups A and C, which was statistically significant (*p* < 0.05). miR-3064-5p and miR-4745-5p expression levels were significantly different among group A, group B, and group C (*p* < 0.05) and were closely correlated with AT-III and FX gene mRNA expression levels, respectively.

**Conclusion:** Differences in heparin sensitivity in patients undergoing cardiac surgery were associated with the mRNA expression of AT-III and FX genes, and the expression levels of miR-3064-5p and miR-4745-5p were found to be closely related to the AT-III and FX gene mRNA, respectively, indicating that miR-3064-5p and miR-4745-5p affect the differences in heparin sensitivity among different individuals by regulating the mRNA expression levels of AT-III and FX genes.

**Clinical Trial Registration:** http://www.chictr.org.cn/abouten.aspx, identifier registration number: ChiCTR-2100047348

## 1 Introduction

Under physiological conditions, coagulation function is systematically and finely regulated to maintain blood flow and is highly alert to any disruption factors of the perfect balance between procoagulation and anticoagulation. However, patients undergoing heart surgery with cardiopulmonary bypass (CPB) require regulation of coagulation function by pharmacological interventions, and this regulation inevitably disrupts the original physiological balance. Abnormal coagulation function in patients undergoing open-heart surgery under CPB is an independent risk factor for increased risk of postoperative bleeding and death.

Heparin is currently the most common anticoagulant used during CPB surgery due to its fast action, high efficacy, easy reversion by protamine, and low cost (H and Jl, 2004). Studies have shown that there is a 3–6 times difference in heparin sensitivity among different individuals (Bartoszko and Karkouti, 2021). In clinical practice, we often ignore this difference and give a standard dose of heparin (3 mg kg^−1^) to all patients, which leads to the disorder of coagulation function. Heparin exerts its anticoagulant effect mainly through antithrombin III (AT-III) and factor X (FX) (Knapik et al., 2012). In a previous study, we have demonstrated that differences in heparin sensitivity in patients undergoing heart surgery were significantly associated with postoperative bleeding and found that differences in heparin sensitivity between individuals were associated with the expression levels of AT-III and FX gene mRNA (Ma, Xu et al., 2020). However, the regulation mechanisms of AT-III and FX gene mRNA expression levels in different individuals are still unclear.

It is known that genes encoding proteins make up only about 1.5% of the genome and most transcripts have little protein-coding potential (Lindblad-Toh, Garber et al., 2011; Ponting and Hardison, 2011; Wang and Chang, 2011; Ma, Xu et al., 2020). However, these noncoding RNAs play a key role in regulating the expression of the target gene (He and Hannon, 2004). Studies have shown that miRNAs play an indispensable role in the regulation of platelet function and secondary hemostasis, and several elements of the coagulation system are regulated by miRNAs, such as platelets, fibrinogen, and tissue factor (Arroyo, de Los Reyes-García et al., 2018). However, it is unclear whether miRNAs are involved in regulating the expression levels of AT-III and FX mRNAs, which affect heparin sensitivity in different individuals. The purpose of this study was to explore the miRNAs associated with AT-III and FX gene expression and to provide a theoretical basis for the realization of individualized anticoagulation strategies.

## 2 Materials and methods

### 2.1 Gene network database prediction

We used the following five databases for miRNA prediction of target genes: Starbase 3.0, TargetScan, miRDB, miRTarBase, and miRWalk. First, we predicted the miRNAs that interacted with AT-III and FX genes in these five online databases, then we cross-checked the five prediction results to obtain prediction targets with higher repetition frequency in the five databases, and finally, five of the common targets with relatively high scores were selected. However, the use of different principles and algorithms for predicting the miRNA target gene in each database resulted in the large difference in the number of miRNAs predicted by the same target gene in different databases.

Among them, the Starbase 3.0 database, miRDB database, and miRTarBase database predicted data mostly from high-throughput or experimentally validated target genes, so fewer miRNAs were predicted. In contrast, the TargetScan database and miRWalk database mainly predicted target genes by the conserved 8mer and 7mer loci matched by miRNA seed regions or other algorithms, so more miRNAs were predicted.

Starbase 3.0: http://starbase.sysu.edu.cn/;
miRDB: http://www.mirdb.org/;
TargetScanHuman: www.TargetScanHuman.org;
miRTarBase: www.microrna.org;
miRWalk: www.uni-heidelberg.de/apps/zmf/mirwalk/index.html;

The specific predicted results are the following.

#### 2.1.1 miRNAs interacting with AT-III genes

**FIGURE 1:**
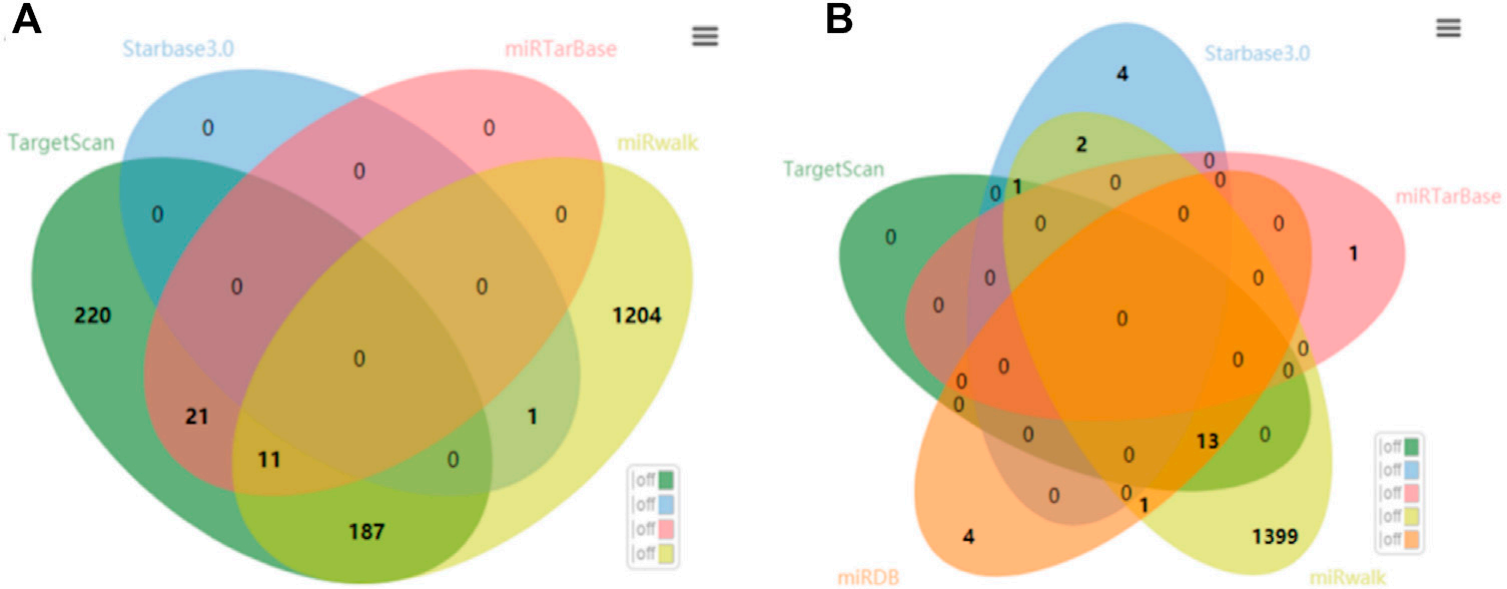
Network prediction Venn diagram of miRNAs interacting with **(A)** AT-III and **(B)** FX genes.

The Starbase 3.0 database predicted 1 miRNA, and the TargetScan database predicted 439 possible binding miRNAs. No miRNAs were predicted to have a targeting relationship with SERPINC1 in the miRDB database. The miRTarBase database predicted 32 possible binding miRNAs, all of which were obtained by second-generation sequencing, and the miRWalk database predicted 1392 possible binding miRNAs. Synthesizing the prediction results of the above five databases, one of the databases did not predict miRNA, and after the intersection of the other four databases, a total of 10 miRNAs were predicted. hsa-miR-6845-3p, hsa-miR-4687-3p, and hsa-miR-7974 with relatively high database scores were selected. hsa-miR-3064-5p and hsa-miR-149-5p with relatively high individual database scores were selected, and a total of five candidate miRNAs were selected. The Venn diagram of the retrieved data is shown in Figure 1A.

#### 2.1.2 miRNAs interacting with factor X genes

The TargetScan and miRWalk databases predicted a total of 739 miRNAs after taking intersections. The other three databases predicted a total of 26 miRNAs (because there were few miRNAs, no intersection was taken). After taking intersections with miRNAs common to the first two databases, a total of 14 miRNAs were predicted. hsa-miR-5047, hsa-miR-6743-3p, and hsa-miR-4745-5p with relatively high database scores were selected, hsa-miR-6776-5p and hsa-miR-4436b-5p with the highest score in the individual database were selected, and a total of five candidate miRNAs were selected. The Venn diagram of the retrieved data is shown in Figure 1B.

### 2.2 Patients

In total, 42 adult patients who underwent cardiac valve replacement surgery in the First Affiliated Hospital of Xinjiang Medical University from September to December 2020 were included in this study. On the basis of the continuity of time, the initial 12 patients were excluded and the later 30 patients met the inclusion criteria and were successfully grouped in the trial. Each patient signed an informed consent form before the study (registration number: ChiCTR-2100047348). The ethical approval for this study was obtained from the Ethics Committee of the First Affiliated Hospital of Xinjiang Medical University (202010226-61).

#### 2.2.1 Inclusion criteria

① Patients 18–65 years of age. ② Patients undergoing elective cardiac valve replacement with CPB. ③ Patients with normal platelet count and preoperative coagulation function. ④ Patients without a history of liver, kidney, and lung dysfunction, and thrombotic or hematological diseases. ⑤ Patients who had signed the informed consent form.

#### 2.2.2 Exclusion criteria

① Patients with preoperative liver or kidney dysfunction. ② Patients with long-term malnutrition or a history of malignant tumors. ③ Patients who had recently undergone surgery. ④ Patients using heparin or taking oral anticoagulants such as aspirin, clopidogrel, warfarin, or ticlopidine during the week before surgery. ⑤ Patients who had a history of long-term hormone use. ⑥ Patients who had previous cardiac surgery or who required emergency surgery. ⑦ Patients with a family history of hereditary disease, history of blood-borne infectious diseases, or hematological diseases. ⑧ Patients with mental illness or inability to cooperate during the study.

### 2.3 Groups

This study followed the grouping method of the previous study (Coakley et al., 2011): as there was no way of knowing a patient’s sensitivity to heparin, all patients were given heparin at a dose of 2.5 mg kg^−1^, the dose was set based on the result of our previous study, which found that 2.5 mg kg^−1^ of heparin enabled 83.7% of CPB surgery patients to achieve the 480 s ACT value required for CPB, and the ACT value after the administration of this dose of heparin facilitated the balanced grouping of the experiment. If the ACT value did not meet the requirement of CPB, a certain dose of heparin would be added until the ACT value was greater than 480 s. Using the minimum effective dose of heparin to meet the requirement of CPB could reduce the intraoperative heparin and protamine dosage, which reduced the risk of postoperative bleeding and heparin-related complications in patients. In addition, we used a 760 s ACT value as the cutoff value. In the past, a 750 s ACT value was the standard for aprotinin in cardiac surgery, and currently, it is considered an excessive anticoagulant. Patients were assigned to one of three groups (A, B, and C) according to the subsequent activated clotting time (ACT).

(1) Group A: hyposensitivity group (ACT < 480 s).
(2) Group B: sensitive group (480 s ≤ ACT ≤ 760 s).
(3) Group C: hypersensitive group (ACT > 760 s).

### 2.4 Protocol

Adult patients underwent cardiac surgery with CPB, and after the induction of anesthesia, an internal jugular vein catheter was inserted. Before heparin was administered, a blood sample (6 ml) was collected from the internal jugular vein of each patient, of which 1 ml was used to determine the baseline ACT and the rest was placed in an EDTA anticoagulant tube and mixed upside down to ensure the anticoagulant effect. The same dose of heparin (2.5 mg kg^−1^) was given after splitting the patient’s sternum; 5 min later, a blood sample (11 ml) was obtained from the internal jugular vein, of which 1 ml was used to determine the ACT and the rest was evenly placed in two EDTA anticoagulant tubes and mixed upside down to ensure the anticoagulant effect. No additional heparin was given if the ACT was more than 480 s. An additional 0.5 mg kg^−1^ of heparin was given if the ACT was less than 480 s; if this was the case, after 5 min, another blood sample was obtained from the internal jugular vein to determine the ACT. Subsequent additional doses of heparin of 0.5 mg kg^−1^ were given as needed until the ACT was more than 480 s. To reverse the effect of heparin at the end of CPB, protamine was given in a 1:1 ratio with the total heparin dose.

The sample was centrifuged at 3000 rpm/min for 5 min at 2 h (4°C). After centrifugation, the upper layer of plasma was carefully aspirated (to avoid aspirating red blood cells and other components) into three clean 1.5 ml cryo-tubes, each about 500 μl, and stored at −80°C. PBMC was separated from the remaining portion according to the human lymphocyte isolation instructions and stored at −80°C. The mRNA of AT-III and FX genes and their predicted miRNAs were centrally detected and analyzed in the PBMC of three groups of patients.

The operative time, CPB time, minimum body temperature during CPB, intraoperative heparin dosage, intraoperative blood loss, and postoperative blood loss (24 h pericardial and thoracic drainages) were recorded.

### 2.5 Outcome measures

(1) Expression levels of AT-III and FX gene mRNA and the levels of predicted 10 miRNAs in patients who underwent cardiac surgery.
(2) Heparin sensitivity index = ACT after initial dose of heparin − ACT baseline/2.5 mg/kg.
(3) Postoperative 24 h blood loss (postoperative thoracic and pericardial drainage) and total blood loss (intraoperative blood loss + postoperative blood loss at 24 h).

Other clinical data

(1) Preoperative coagulation function indicators: international standard ratio, ACT, and prothrombin time.
(2) Observation indicators: intraoperative blood loss, total blood loss, total heparin dose, operation time, CPB time, and CPB lowest temperature.

### 2.6 Assays and equipment for outcome measures

AT-III activity was determined in the test center of our hospital using chromogenic substrate assay (ACL-TOP automated coagulation analyzer with manufacturer-supplied reagents [lot#: NO796933]; Werfen Group, Spain), in strict accordance with the instructions for use.

ACT was determined using an ACT analyzer (ACTⅡplus^TM^; Medtronic, United States) with manufacturer-supplied tubes.

The mRNA levels of AT-III and factor X were determined with real-time fluorescent quantitative PCR (RT-PCR), and the RT-PCR primers were designed as follows (Tables 1, 2).

**TABLE 1:** Primer information table for fluorescence quantitative AT-III and FX gene mRNA detection.

Primer	Sequence, (5′ to 3′)	Product size
SERPINC1-F	AGA​GCG​GCC​ATC​AAC​AAA​TG	131 bp
SERPINC1-R	TTC​CAC​AGG​CCC​TTG​AAG​TAA	
F10-F	GCT​CGG​GGA​AAG​TCT​GTT​CAT	129 bp
F10-R	CAG​GTC​TCT​TCC​ATG​CAC​TCT​C	
GAPDH_F	GGA​GCG​AGA​TCC​CTC​CAA​AAT	197 bp
GAPDH_R	GGC​TGT​TGT​CAT​ACT​TCT​CAT​GG	

**TABLE 2:** Primer information table for fluorescent quantitative miRNA detection.

Primer	Sequence, (5′ to 3′)
hsa miR 6845-3P	AGCCTCTCCTCCCTGT
hsa miR 4687-3P	GCAGTGGCTGTTGGAG
hsa miR 7974	GGCTGTGATGCTCTCCT
hsa miR 3064-5P	CGCAGTCTGGCTGTTGT
hsa miR 149-5P	CTGGCTCCGTGTCTTC
hsa miR 5047	TGCAGCTGCGGTTG
hsa miR 6743-3P	GCCGCTCTTCTCCCT
hsa miR 4745-5P	GCAGTGAGTGGGGCTC
hsa miR 6776-5P	CAGTCTGGGTGCAGTG
hsa miR 4436B-5P	CACTTCTGCCTGCCCT
hsa U6	CTCGCTTCGGCAGCACA

Equipment and reagent for RT-PCR: (PCR detection equipment: Bio-Rad, MyCycler Thermal Cycler, United States); mRNA levels of AT-III and FX (Reagent: TRIzol^®^ Reagent; Manufacturer: Invitrogen, Cat. No.: 15596026); miRNA was determined with fluorescent quantitative PCR kit (dye method) (Manufacturer: Shanghai Shenggong, Cat. No.: B532461, Lot No.: G319KA4896). All are tested centrally in OE (OE Bio Tech, Shanghai, China).

### 2.7 Statistical analysis

We did not use a randomized, double-blind trial design because patients were divided into different groups according to ACT values after the initial heparin dose. For the data that did not conform to the normal distribution, the measurement data were expressed as median and interquartile deviation, namely, M (P25, P75). For the data that did conform to the normal distribution, the measurement data were expressed as the mean ± SD ( $\bar{x}\pm s$ ). In addition, the count data were all expressed as frequency or percentage. Kruskal–Wallis test was performed for among-group comparisons, and categorical data were assessed using chi-square (χ^2^) tests. SPSS 18.0 software was used for the statistical analysis, and a value of *p* less than 0.05 was considered statistically significant.

## 3 Results

### 3.1 Study population

**FIGURE 2:**
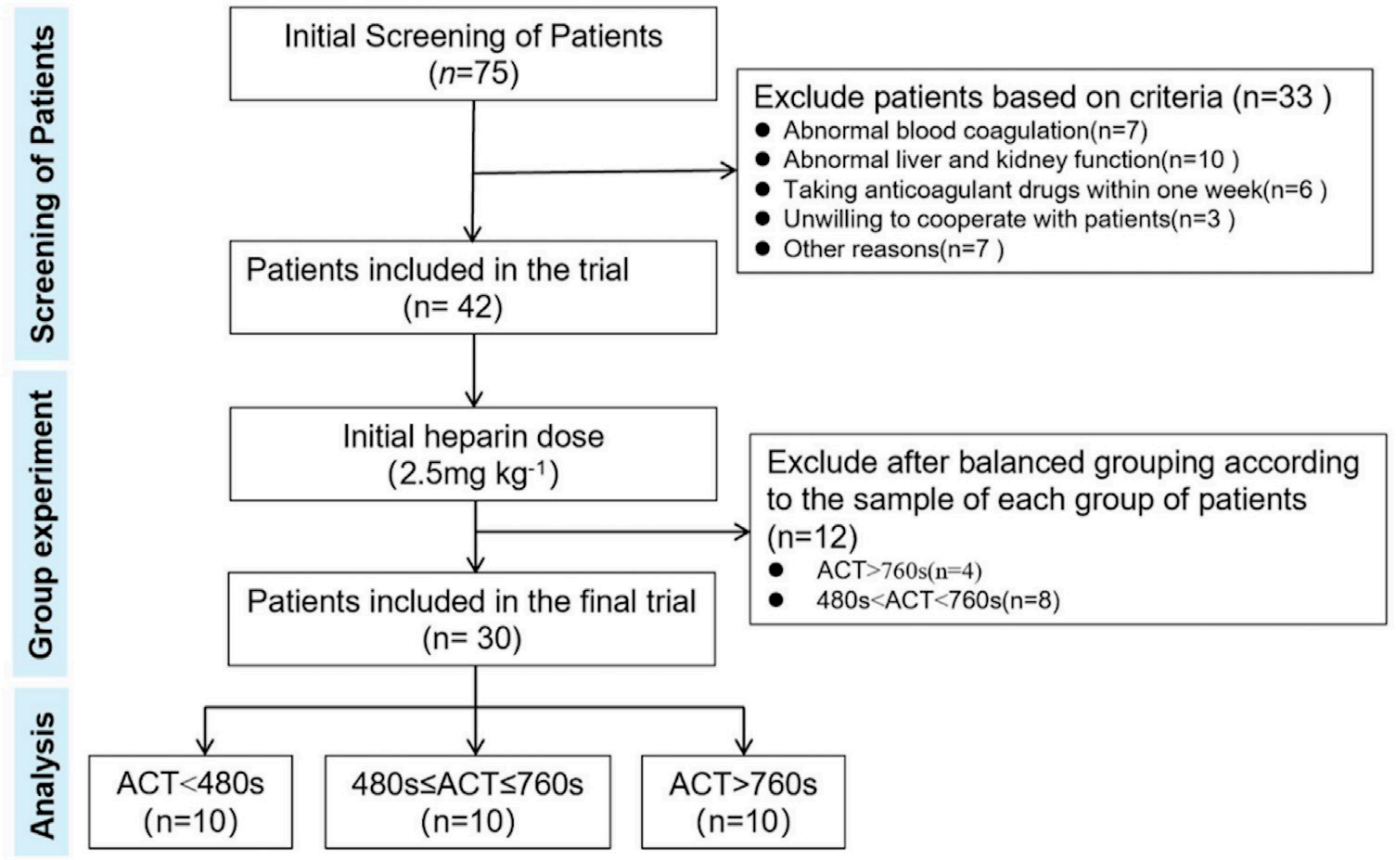
Flowchart for the inclusion of patients.

From the 75 patients screened initially (Figure 2), 33 were excluded according to the inclusion criteria. After the initial dose of heparin (2.5 mg kg^−1^) was given to 42 patients who met the inclusion criteria, 10 patients were actually included in each group in order to achieve balanced grouping, and a total of 30 patients were finally included. Patients with abnormal liver and kidney function, abnormal coagulation, and recent anticoagulant use were not included in the study because the results involved coagulation indicators, and the principle of balanced grouping was adopted to reduce confounding factors for subsequent studies after the first heparin dose was given. They were divided into three groups: ACT<480 s, 480 s≤ACT≤760 s, and ACT>760 s. There was no significant difference in perioperation characteristics (including age, gender, weight, BMI, preoperative coagulation, complications, operation, and CPB time among the groups) (Table 3).

**TABLE 3:** Perioperation characteristics.

Variable	Group A, *n* = 10 ACT<480s	Group B, *n* = 10 480s≤ACT≤760s	Group C, *n* = 10 ACT>760s	*p* Value
Pre-operative				
Sex (male%)	5 (50)	3 (30)	5 (50)	0.59
Age (years)	53.5 (43.7,60.5)	47.5 (38.7,56.0)	46.0 (37.2,53.2)	0.33
Weight (kg)	66.0 (60.0,85.5)	67.0 (59.5,87.0)	77.5 (60.2,82.7)	0.83
BMI (kg m^−2^)	22.5 (20.0,28.7)	24.5 (22.5,27.7)	27.0 (20.8,30.0)	0.71
Coagulation function index				
APTT(s)	30.3 (30.2,30.6)	29.8 (28.7,30.5)	30.5 (30.0,30.7)	0.26
PT(s)	11.5 (11.4,11.5)	11.5 (11.5,11.5)	11.5 (11.5,11.5)	0.54
INR	1.0 (1.0,1.2)	1.1 (1.0,1.2)	1.1 (1.0,1.2)	0.71
AT-III activity (%)	81.4 (79.7,84.2)	84.4 (81.7,87.8)	84.5 (81.9,86.3)	0.10
Platelet count (109L^−1^)	204.5 (144.5,278.5)	186.0 (154.0,248.3)	215.5 (131.3,241.5)	0.93
Initial haemoglobin (g l^−1^)	138 (126.3,149.8)	133.0 (116.5,147.0)	127.5 (119.5,133.8)	0.42
Combined diseases				
Diabetes (%)	0	0	1 (10)	0.11
Hypertension (%)	3 (30)	4 (40)	1 (10)	0.24
Intra-operative				
Cardiac surgery type				
Mitral valve replacement (%)	4 (40)	2 (20)	3 (30)	0.63
Aortic valve replacement (%)	2 (20)	2 (20)	3 (30)	0.84
Tricuspid valve replacement (%)	2 (20)	3 (30)	2 (20)	0.84
Combined valve replacement (%)	2 (20)	3 (30)	2 (20)	0.84
Operation time (min)	217.5 (180.0,240.0)	217.5 (210.0,225.0)	225 (180.0,240.0)	0.97
CPB time (min)	81.5 (63.8,89.3)	75.0 (68.5,81.8)	72.0 (68,185.8)	0.88
CPB lowest temperature (°C)	33.5 (32.6,33.9)	33.5 (32.8,34,3)	33.1 (32.6,33.6)	0.06
Postoperative				
ICU length of stay (h)	81.0 (37,123.5)	70.0 (53.5,95.5)	71.5 (46.5,120.0)	0.99
Hospital length of stay (days)	22.5 (19.8,26.3)	20.5 (19.0,23.0)	21.5 (19.8,27.0)	0.66

### 3.2 Heparin sensitivity and postoperative bleeding in the three groups

Comparing the amount of blood loss during operation and 24 h after the operation, we found that the heparin sensitivity index of group A to group C increased successively, with a significant difference (*p* < 0.05). It was found that group B had the least postoperative bleeding, with a significant difference compared with the other two groups (*p* < 0.05). The total doses of heparin and protamine in group A were apparently higher than those in groups B and C (*p* < 0.05), and there was no difference in baseline ACT among groups (Table 4).

**TABLE 4:** Heparin sensitivity and bleeding.

Variable	Group A, *n* = 10 ACT<480s	Group B, *n* = 10 480s≤ACT≤760s	Group C, *n* = 10 ACT>760s	*p* Value
Baseline ACT(s)	161.0 (143.3,167.3)	163.0 (141.5,186.0)	159.5 (150.8,172.8)	0.73
Total heparin dosage (mg)	210.0 (200.0,240.0)	180.0 (190.0,200.0)	185.0 (175.0,200.0)	0.01
Total protamine dosage (mg)	240.0 (207.5,246.3)	197.5 (185.0,210.0)	210.0 (180.0,232.5)	0.01
ACT after the initial dose of heparin (s)	430.5 (396.3,469.3)	596.5 (529.5,721.3)	958.5 (905.5,999.0)	0.00
Heparin sensitivity index (s/mg^−1^ kg^−1^)	138.8 (114.8,150.0)	217.0 (191.8,174.8)	393.3 (373.4,418.9)	0.00
Intra-operative blood loss (ml)	500.0 (437.5,600.0)	450.0 (400.0,500.0)	475.0 (400.0,562.5)	0.48
24-h postoperative bleeding (ml)	410.0 (375.0,500.0)	355.0 (315.0,402.5)	415.0 (400.0,435.0)	0.02
Total blood loss (ml)	910.0 (852.5,1002.5)	820.0 (737.5,902.5)	905.0 (850.0,970.0)	0.06

Data are median (IQR) or n (%); ACT, activated clotting time.

### 3.3 Heparin sensitivity and AT-III and factor X miRNA in the three groups

**FIGURE 3:**
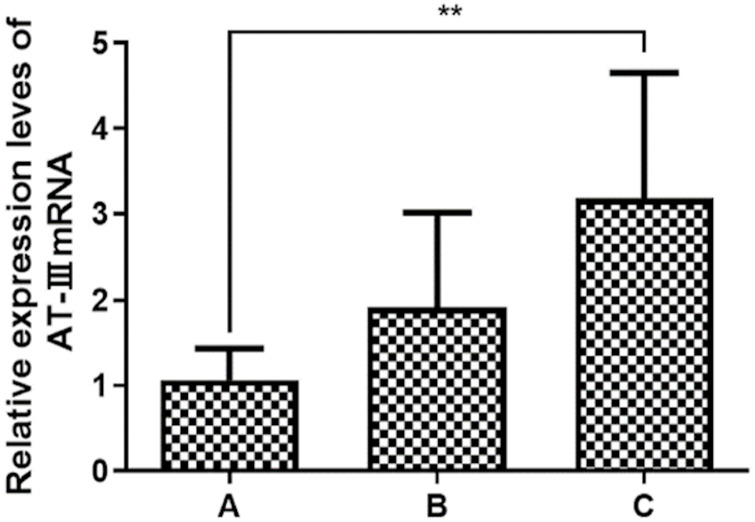
Expression levels of AT-III miRNA. *p<0.05 vs. A; **p<0.01 vs. A.

**FIGURE 4:**
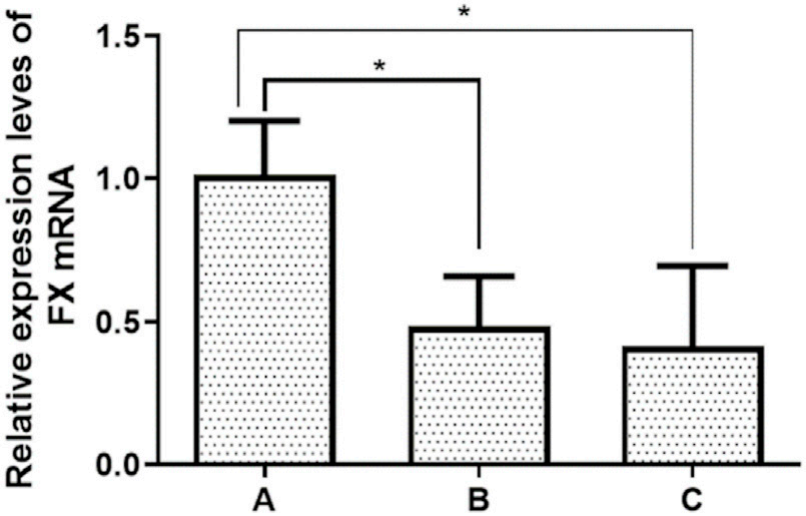
Expression levels of FX miRNA. *p<0.05 vs. A; **p<0.01 vs. A.

The results of the detection of AT-III and FX gene mRNA in PBMC of patients showed that heparin sensitivity was positively correlated with the level of AT-III mRNA, Pearson correlation test results showed a positive correlation, and there was a statistically significant difference between group C and groups A and B (*p<*0.05). Heparin sensitivity was negatively correlated with the level of FX gene mRNA, Pearson correlation test results showed a negative correlation, and there was a statistically significant difference between group A and groups B and C (*p* < 0.05) (Table 5, Figures 3, 4).

**TABLE 5:** Correlation of AT-III and FX with heparin sensitivity.

	AT-III	FX
*R value*	0.567	−0.624
*p value*	0.001	0.001

### 3.4 Correlation between AT-III gene mRNA and corresponding miRNA in the three groups

**FIGURE 5:**
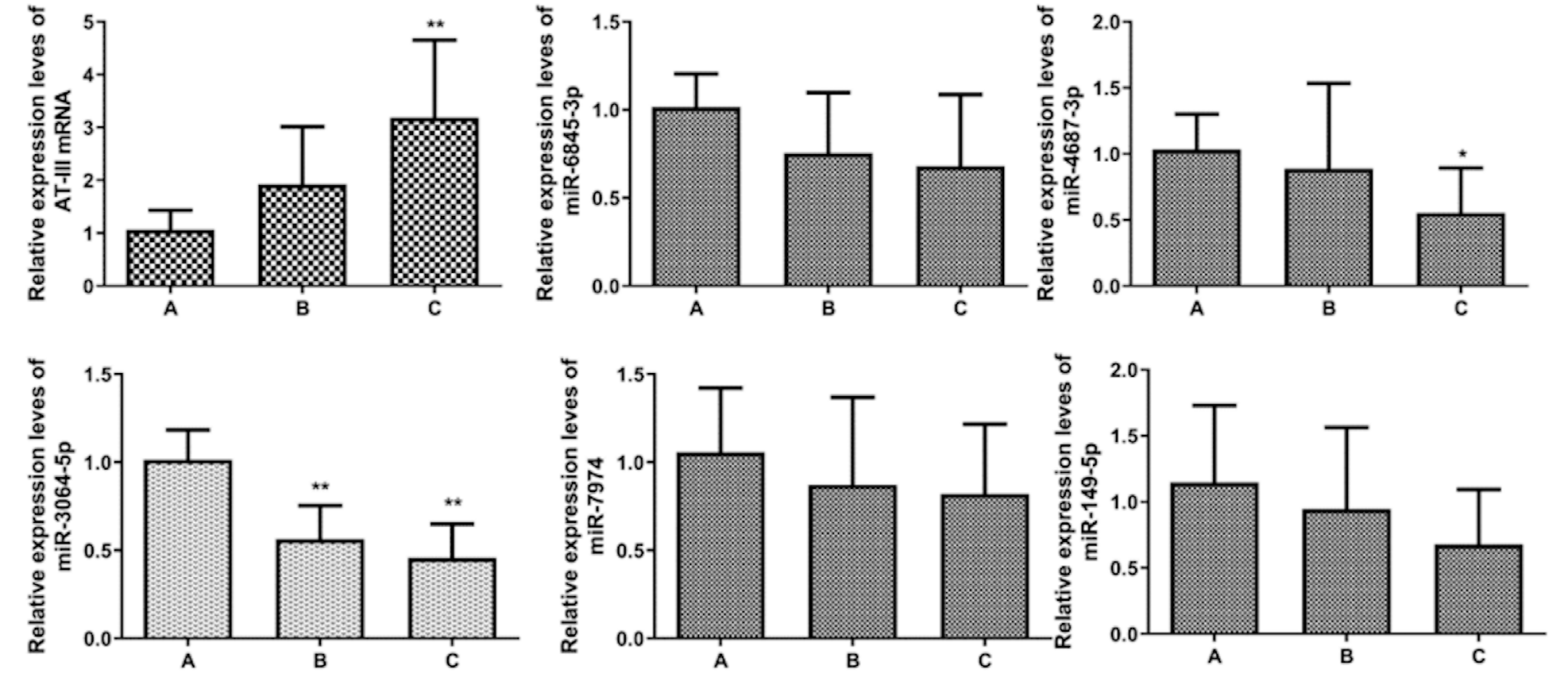
AT-III miRNA and miRNA qRT-PCR detection. *p<0.05 vs. A; **p<0.01 vs. A.

RT-PCR detected the expression levels of five miRNAs associated with AT-III gene mRNA, and the results showed that the expression levels of the five miRNAs were gradually decreasing from group A to group C. Pearson correlation test showed that all five miRNAs were negatively correlated with the expression level of AT-III, and only miR-3064-5p showed a statistically significant difference among group A, group B, and group C (*p* < 0.05). The results showed that miR-3064-5p was closely related to AT-III gene mRNA, and there was a negative correlation between the two, suggesting that miR-3064-5p was involved in the regulation of the AT-III gene mRNA expression level (Tables 6, 7; Figure 5).

**TABLE 6:** Expression levels of AT-III miRNA gene and corresponding miRNA in PBMC of patients ( $\bar{x}\pm s$ , *n* = 10).

Group	Group A	Group B	Group C
SERPINC1	1.065 ± 0.371	1.922 ± 1.092	3.187 ± 1.466△
miR-6845-3p	1.015 ± 0.189	0.754 ± 0.344	0.680 ± 0.407
miR-4687-3p	1.032 ± 0.269	0.888 ± 0.647	0.552 ± 0.341
miR-7974	1.056 ± 0.366	0.870 ± 0.499	0.819 ± 0.398
miR-3064-5p	1.013 ± 0.170	0.562 ± 0.193△	0.456 ± 0.194△
miR-149-5p	1.145 ± 0.584	0.944 ± 0.620	0.678 ± 0.416

△ compared with group A, *p* < 0.05; ▲ Compared with group B, *p* < 0.05.

**TABLE 7:** Correlation between AT-III and corresponding miRNA.

	miR-6845-3P	miR-4687-3P	miR-7974	miR-3064-5P	miR-149-5P
*R*	−0.248	−0.445	−0.241	−0.572	−0.247
*p*	0.187	0.014	0.200	0.001	0.188

### 3.5 Correlation between factor X gene mRNA and corresponding miRNA in the three groups

**FIGURE 6:**
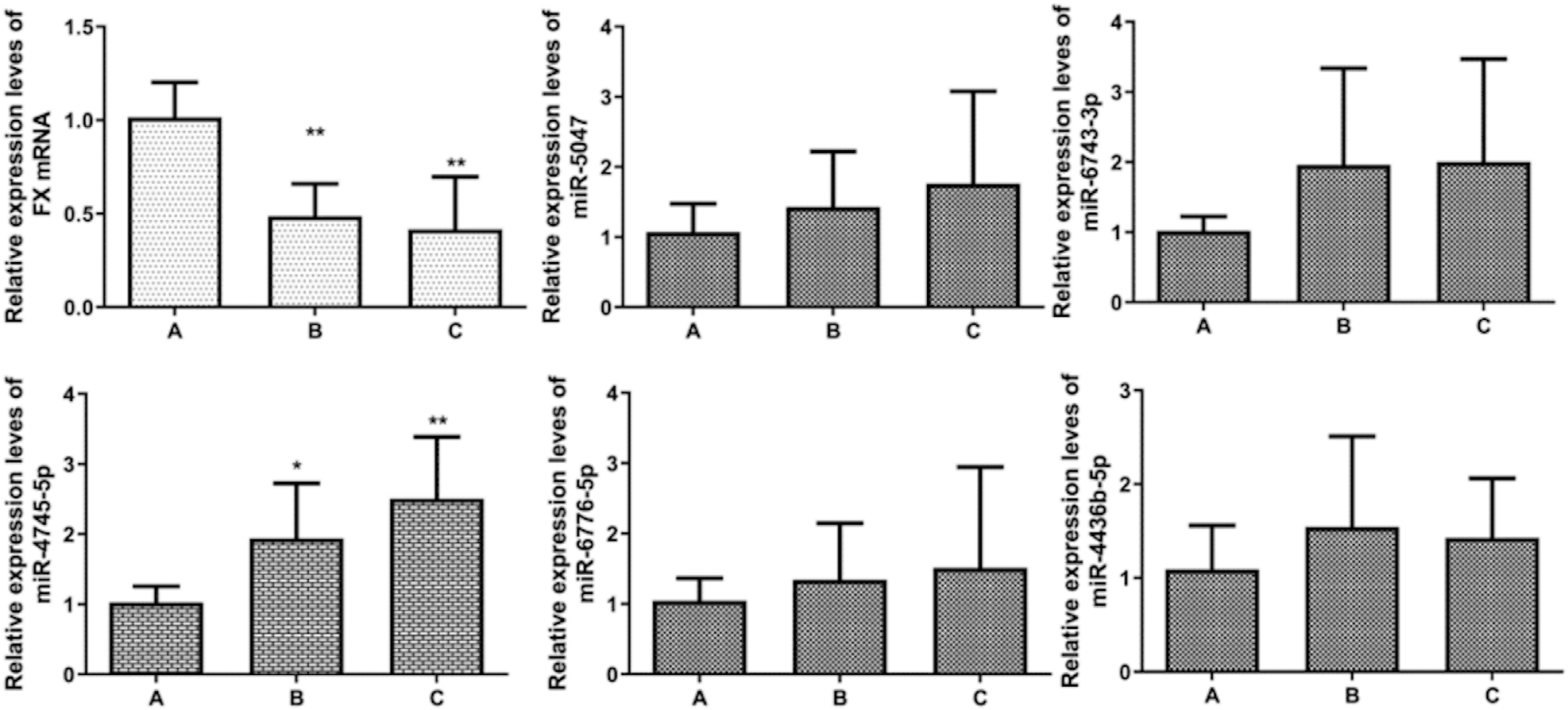
FX miRNA gene and miRNA qRT-PCR detection. *p<0.05 vs. A; **p<0.01 vs. A.

RT-PCR detected the expression levels of five miRNAs associated with the FX gene mRNA, and it was found that the expression levels of the five miRNAs showed a gradual increase from group A to group C. Pearson correlation test showed that all five miRNAs were positively correlated with the expression level of FX, and only miR-4745-5p showed a statistically significant difference among group A, group B, and group C (*p* < 0.05). The results showed that miR-4745-5p was involved in the regulation of the FX gene mRNA expression level (Tables 8, 9; Figure 6).

**TABLE 8:** Expression levels of FX miRNA gene and corresponding miRNA in PBMC of patients ( $\bar{x}\pm s$ , *n* = 10).

Groups	Group A	Group B	Group C
F10	1.015 ± 0.188	0.485 ± 0.175	0.417 ± 0.281
miR-5047	1.072 ± 0.403	1.428 ± 0.792	1.761 ± 1.321
miR-6743-3p	1.018 ± 0.208	1.958 ± 1.379	2.003 ± 1.468
miR-4745-5p	1.025 ± 0.234	1.937 ± 0.789△	2.506 ± 0.881△
miR-6776-5p	1.045 ± 0.321	1.342 ± 0.807	1.511 ± 1.439
miR-4436b-5p	1.091 ± 0.469	1.543 ± 0.969	1.428 ± 0.636

**TABLE 9:** Correlation between FX and corresponding miRNA.

	miR-5047	miR-6743-3P	miR-4745-5P	miR-6776-5P	miR-4436b-5P
*r*	0.206	0.284	0.625	0.300	0.176
*p*	0.274	0.128	0.001	0.107	0.351

## 4 Discussion

In this study, we first screened out five miRNAs with a high correlation with AT-III and five miRNAs with a high correlation with FX using online database prediction, and then, patients undergoing heart valve surgery were grouped according to their different heparin sensitivities after the first heparin dose was given (2.5 mg kg^−1^). The results showed that the different heparin sensitivity was related to the mRNA expression levels of AT-III and FX genes, and the postoperative bleeding was related to heparin sensitivity. In addition, the expression levels of miR-3064-5p and miR-4745-5p were found to be closely related to the mRNA of AT-III and FX, respectively, indicating that miR-3064-5p and miR-4745-5p may affect the differences in heparin sensitivity among individuals by regulating the mRNA expression level of AT-III and FX genes.

Studies have shown that using the same dose of heparin increases the risk of reoperation and perioperative death in patients undergoing heart surgery (He and Hannon, 2004). It has been reported that low doses of heparin can significantly reduce postoperative bleeding and the blood transfusion requirements compared to standard heparin dosage (Nilsson, Scicluna et al., 2012), and individualized heparin anticoagulation management can significantly improve clinical outcomes (Pai and Crowther, 2012; Vonk, Veerhoek et al., 2014). In this study, the postoperative bleeding of group B was significantly less than that of the other two groups, and the use of heparin and protamine in group A was significantly higher than that of the other two groups, indicating that high-dose heparin and protamine and excessive anticoagulation may be related to postoperative bleeding. Therefore, how to achieve individualized and precise heparin anticoagulation management is an extremely important scientific question for patients undergoing cardiovascular surgery.

After the use of heparin, AT-III inactivates thrombin and FXa 1000 and 3500 times faster, respectively (Hao, Xu et al., 2019). Whole blood ACT is a standard index for measuring the anticoagulant effect of heparin (Coakley et al., 2011). Sensitivity of heparin in different individuals is expressed by the sensitivity index. As can be seen from the calculation formula of heparin sensitivity index (ACT after the initial dose of heparin − ACT baseline/heparin dose per kg body weight), it reflects the relationship between heparin dose and anticoagulant effect (ACT value) (Finley and Greenberg, 2013). The differences in heparin sensitivity are the result of the combination of the genetic diversity of different individuals and the complex clinical environment. Clinical factors such as hemodilution, hypothermia, and abnormal platelet function all have an impact on heparin sensitivity (Sniecinski and Levy, 2015). In the present study, there was no statistical significance in coagulation function, AT-III activity, and platelet count between the groups. Therefore, the ACT after the same initial dose of heparin represents the heparin sensitivity index. Our previous study has confirmed that heparin sensitivity is related to the mRNA expression levels of AT-III and FX genes (Ma, Xu et al., 2020), and the results of the present study reconfirmed the previous findings. The clinical confounders in this study were all controlled at the same level, so the factors affecting the mRNA expression levels of AT-III and FX genes become important.

miRNAs are small endogenous single-stranded noncoding RNA molecules, consisting of about 22 nucleotides and ranging from 18–25 nt in length, present in eukaryotic cells. They can regulate approximately 60% of mammalian protein-coding genes by binding to specific complementary sequences in the noncoding region at the 3′UTR end of mRNA to mediate the degradation of mRNA or to repress protein translation (Eyileten, Wicik et al., 2018). miRNAs are highly conservative, sequential, and tissue-specific and can regulate specific physiological functions of organisms, playing a key role in growth and disease development of organisms (He, He et al., 2007; Nelson, Wang et al., 2008; Greco, Gorospe et al., 2015; Bijak, Dzieciol et al., 2016). Each miRNA can control multiple target genes, and a single gene can be controlled by more than one miRNA. Therefore, this complex regulatory network can regulate the expression of multiple genes through a single miRNA and can specifically regulate the expression of a single gene through the binding of multiple miRNAs (Volinia, Galasso et al., 2010).

At present, mathematical modeling, bioinformatics analysis, and experimental validation based on big data platforms have been widely used to study the physiological role of miRNA in regulating target protein-related cells. Our study used five online databases to predict five miRNAs each highly correlated with AT-III and FX genes. Real-time quantitative PCR (RT-PCR) has become the gold standard for quantitative detection of nucleic acid molecules due to its high sensitivity, repeatability, and specificity (Jiang, Lee et al., 2005). In this study, the expression levels of AT-III and FX gene mRNAs and corresponding miRNAs in the three groups were detected by RT-PCR. The results showed that the expression level of miR-3064-5p showed a statistically significant difference between groups and was negatively correlated with the expression level of AT-III mRNA. In a similar way, miR-4745-5p was negatively correlated with the mRNA expression level of FX. After analysis, it was found that miR-3064-5p and miR-4745-5p caused the difference in heparin sensitivity in different individuals by regulating the mRNA expression of AT-III and FX genes, respectively.

The results of this study provide a new idea for the problems of heparin sensitivity and heparin resistance in clinical heparin use. If further studies reveal the exact relationship between miR-3064-5p and miR-4745-5p and heparin sensitivity, it will be possible to predict the degree of heparin sensitivity before cardiac surgery and to make a precise heparin regimen.

The study of miRNA mainly consists of two stages, namely, screening and individual validation, and the lack of validation is a shortcoming as only screening was completed in this study. Therefore, it needs to be further verified in animal tests and then verified again in clinical practice using gene chip technology.

To sum up, in this study, through online database screening and clinical trials, it was found that the difference in heparin sensitivity in cardiac surgery patients was related to the expression levels of AT-III and FX gene mRNA. In addition, the expression levels of miR-3064-5p and miR-4745-5p were closely related to the expression levels of AT-III and FX gene mRNA, respectively. The results showed that miR-3064-5p and miR-4745-5p caused the difference in heparin sensitivity in different individuals by regulating the mRNA expression of AT-III and FX genes. This study provides some theoretical basis for further individualized dosing management of heparin, and further experimental verification is needed.

There are some shortcomings in our experiment. First, we used the method of taking the concatenation of the Starbase 3.0 database, miRDB database, and miRTarBase database, and the intersection of the TargetScan database and miRWalk database, the results of both were then intersected to identify the candidate miRNAs, and we tend to select the miRNAs with experimental validation. This has narrowed the scope of our validation and has increased the probability of successful validation. However, the selection of experimentally validated miRNAs does miss some potential miRNAs, which is an area for further study in subsequent experiments. Second, the present study only contains the online database prediction and clinical validation part, no basic experimental research has been conducted, and its specific mechanism will be the subsequent research content and direction of our research group.

## Data Availability

The raw data supporting the conclusion of this article will be made available by the authors, without undue reservation.
